# Refractory Metals and Oxides for High-Temperature Structural Color Filters

**DOI:** 10.1021/acsami.2c14613

**Published:** 2022-12-06

**Authors:** Margaret A. Duncan, Landin Barney, Mariama Rebello Sousa Dias, Marina S. Leite

**Affiliations:** † Department of Materials Science and Engineering, UC Davis, 1 Shields Ave, Davis, California 95616, United States; ‡ Department of Physics, University of Richmond, 138 UR Drive, Richmond, Virginia 23173, United States

**Keywords:** refractory metals, high-temperature photonics, structural colors, dielectric functions, *in situ* ellipsometry

## Abstract

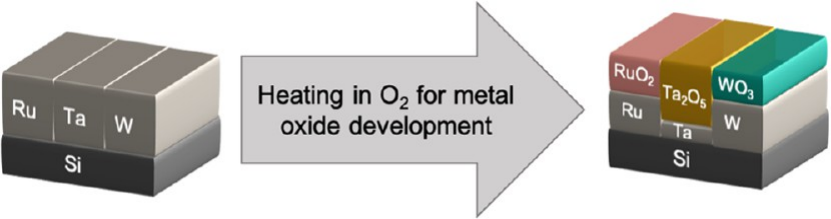

Refractory metals have recently garnered significant interest as options for photonic applications due to their superior high-temperature stability and versatile optical properties. However, most previous studies only consider their room-temperature optical properties when analyzing these materials’ behavior as optical components. Here, we demonstrate structural color pixels based on three refractory metals (Ru, Ta, and W) for high-temperature applications. We quantify their optical behavior in an oxygenated environment and determine their dielectric functions after heating up to 600 °C. We use *in situ* oxidation, a fundamental chemical reaction, to form nanometer-scale metal oxide thin-film bilayers on each refractory metal. We fully characterize the behavior of the newly formed thin-film interference structures, which exhibit vibrant color changes upon high-temperature treatment. Finally, we present optical simulations showing the full range of hues achievable with a simple two-layer metal oxide/metal reflector structure. All of these materials have melting points >1100 °C, with the Ta-based structure offering high-temperature stability, and the Ru- and W-based options providing an alternative for reversible color filters, at high temperatures in inert or vacuum environments. Our approach is uniquely suitable for high-temperature photonics, where the oxides can be used as conformal coatings to produce a wide variety of colors across a large portion of the color gamut.

## Introduction

Structural color refers to any process where hue is generated utilizing micro- or nanostructured surfaces. These surfaces interact with incident light, changing its reflection or adding absorption peaks, which can result in the production of vibrant colors.^1−4^ The shades formed by this process are often far more stable than traditional ink printing options and can offer further printing precision given the microscopic or nanoscopic scale of the fabrication. Many modern attempts at creating artificial structural color can produce vivid, robust shades, but rely on complex metasurfaces^5^ or many-layer geometries designed to exploit Fabry–Perot resonances.^6,7^ Structural colors are quickly growing in their usage, and have applications in sensing,^8−11^ anticounterfeit technology,^12−15^ solar selective absorbers for photovoltaics,^16,17^ and heat-resistant coatings.^18^

In an all-thin-film design, both metallic and dielectric materials are needed to fulfill the thin-film interference conditions required for forming reflective color filters [DOI: 10.1002/adom.202200159]. Yet, most previous structural color designs would not be capable of withstanding high-temperature treatment because the materials commonly used present limited thermal properties (e.g., low melting point, high thermal expansion, etc.). However, several refractory metals and their oxides offer melting points above 1100 °C, representing, thus, a promising platform for generating structural colors that can be used under extreme high-temperature conditions. As an example, prior works using W and Mo oxides for this purpose have used nonstoichiometric metal oxides fabricated *via* sputtering on a glass substrate^17^ or on a different metallic substrate like Al or Cu.^19^

In this work, we circumvent the thermal limitations imposed by the modest melting point of coin-age metals (Au, Ag, Cu) by realizing a scalable geometry utilizing refractory metals and their oxides for proof-of-concept structural color printing that can operate at high temperatures. Our material selection entails refractory metals with melting point >1100 °C, significantly superior to the coin-age metals. While this class of material has been underexplored for photonics thus far, we show that their optical behavior (i.e., permittivity) is very suitable for devices in the visible range of the electromagnetic spectrum. By controlling *in situ* the oxidation of Ru, Ta, and W thin films, we attain an alternative route for tailoring the spectrum. We fabricate structural color filters that produce vivid colors ranging from dark yellow to light pink and cyan, by performing a controlled heating treatment while measuring the samples *in situ* with ellipsometry. The hues result from interference between the incoming and outgoing light, which changes depending on the thickness of the MO*_x_* layer and the dielectric function of the metal. The colors are obtained by submitting each refractory metal to a thermal treatment at 600 °C in an oxidizing environment. Oxygen diffusion within these refractory metals leads to a dual-layer dielectric/metal structure that enables light interference, which, in turn, gives rise to the primary printing colors. These hues are angle-insensitive up to 75° for RuO_2_, and up to 65° for Ta_2_O_5_ and WO_3_. Furthermore, optical simulations of similar device structures show that a large portion of the color gamut can be reached simply by changing the thickness of the metal oxide layer. The permittivity for all metals and their oxides has been consistently modeled using general oscillators, and these data are made fully available to enable other researchers to use them when designing optical building blocks for additional high-temperature applications. Our results illustrate how refractory metals can be implemented for color printing, with the flexibility of selecting either static or reversible responses at temperatures beyond 1000 °C, depending on material and environment. Given the thermal stability of Ta_2_O_5_ in inert environments,^20^ these structural color systems would be ideal optical coatings for space applications. Alternately, using further oxidation of all three refractory-metal-based structural color systems in an oxygen-rich environment, these structures could be implemented as simple, yet highly sensitive oxygen sensors. Materials that present suitable optical properties (low loss) *and* are chemically controllable at high temperatures have been increasingly sought after recently due to their potential usage in ultrahigh-temperature, extreme conditions. In turn, these findings are launching refractory-metal oxides as a class of material for ultrahigh-temperature photonics.

## Results and Discussion

**Figure 1:**
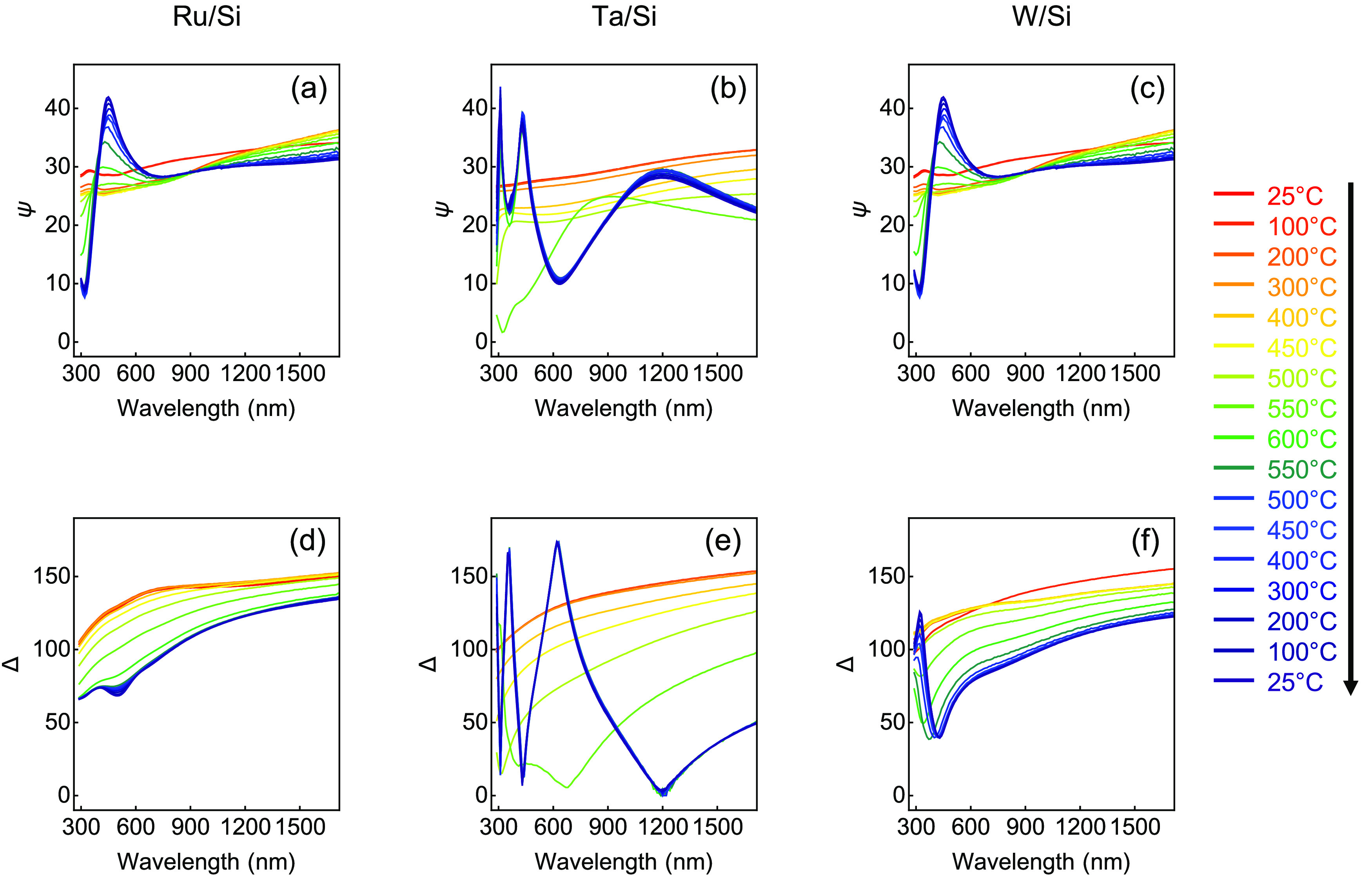
High-temperature treatment. Ellipsometric parameters (a–c) Ψ and (d–f) Δ. *In situ* optical measurements through high-temperature cycle for (a, d) Ru, (b, e) Ta, and (c, f) W on Si substrates. With these two parameters, we can characterize the optical properties of these materials as they change with increasing temperature. All curves shown are at an angle of 70° from normal incidence. The black arrow shows the order of measurements.

To obtain refractory metal oxides, we heat the samples to 600 °C in an oxidizing environment (mixture of air and Ar) while measuring their optical properties using *in situ* spectroscopic ellipsometry. We use a ramping rate of 3 °C min^–1^, stopping at each 100 °C point for 22 min with additional steps of 50 °C above 400 °C to allow the samples to thermalize (see Figure S1 in the Supporting Information for temperature profile). Figure 1 shows the *in situ* ellipsometry measurements of the refractory metals from room temperature throughout the high-temperature cycling process. The ellipsometric parameters Ψ and Δ refer, respectively, to the ratio of the amplitude of the reflected *s*- and *p*- polarized light, and the phase difference between the reflected *s*- and *p*-polarized light.^21,22^ Together, they characterize the reflection behavior from the surface of our system. All three films show stark changes in their reflective properties beginning at 500 °C, the temperature at which oxygen will begin to diffuse into the bulk of the three metals.^23−26^ Clear peaks develop at 500 °C and continue to increase in magnitude for the remainder of the temperature ramp process, coinciding with reflective interference due to the growth of the corresponding dielectric layers.^27,28^ This alteration is evidenced by a color change in the reflection spectrum (see Figure S1 in the Supporting Information for sample photographs). The location of these peaks shifts slightly toward higher wavelengths as the temperature increases, due to the increasing thickness of the dielectric layer. Given this knowledge, the ability to perform an *in situ* characterization of the samples *via* ellipsometry allows for the precise control of the thickness of the oxide layer and their optical properties.

**Figure 2:**
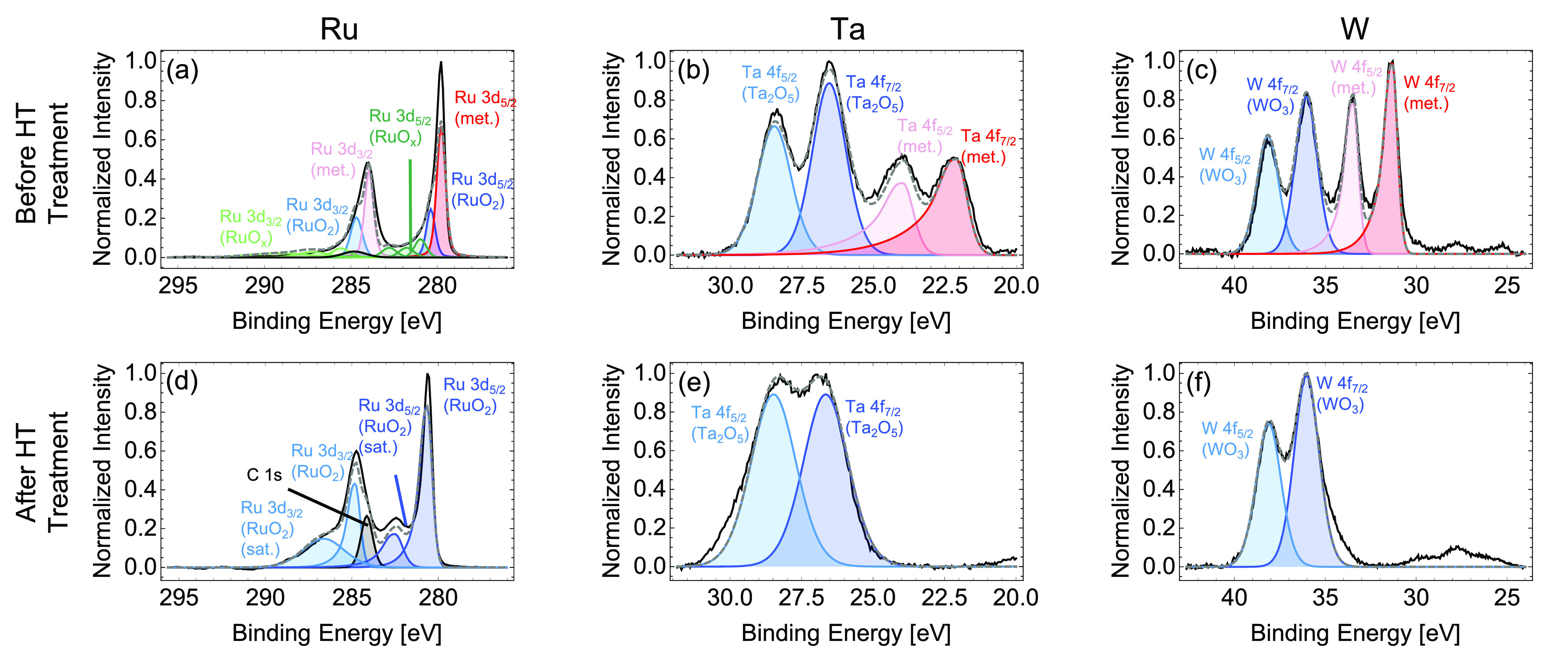
Chemical composition analysis. X-ray photoelectron spectroscopy (a–c) before and (d–f) after high-temperature treatment for Ru, Ta, and W. All three samples suffer 100% surface oxidation after high-temperature treatment. In all plots, the black solid line and gray dashed line refer to raw data and their respective fits using the contributions of all peaks in blue and red, respectively. The chemical compositions of each constituent peak are shown in the plots for reference.

We use X-ray photoelectron spectroscopy (XPS) to discern the specific composition of the oxide layer and analyze any change in surface chemistry with heat treatment. Figure 2 shows the XPS spectra before and after temperature treatment, where the measured and fitted data are presented in black solid line and gray dashed line, respectively. In Figure 2a–c, we see the signature of thin native oxide layers in all samples before high-temperature treatment. This oxide layer is less than 10 nm thick, given the known penetration depth limitation of XPS.^29^ This aligns well with previous literature sources, which found thicknesses of native oxides for all three materials to be less than 2 nm at room temperature.^23,26,30^ For the pristine samples, the XPS data are fitted by a combination of the metals and their oxides in blue and red, respectively. Upon temperature cycling, the intrinsic oxide layers develop by slowly consuming the metals. From Figure 2d–f, the pure elemental peaks are no longer present, indicating a metal oxide thickness of at least 10 nm. Compared to the literature, we determine the stoichiometry of the oxide layers to be RuO_2_^31,32^ for Ru, Ta_2_O_5_^33^ for Ta, and WO_3_^34^ for W.

**Figure 3:**
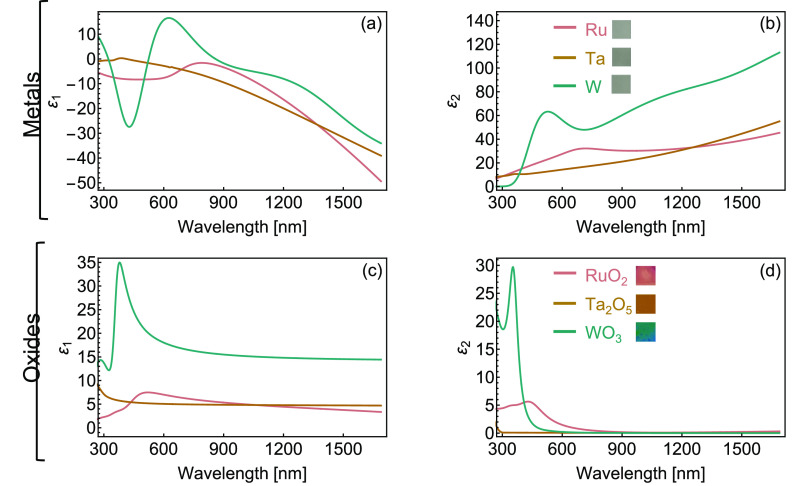
Optical behavior of refractory metals and their oxides. (a) Real (ε_1_) and (b) imaginary (ε_2_) components of the dielectric function of Ru (pink), Ta (yellow), and W (cyan) thin films, showing metallic behavior. (c) Real and (d) imaginary components of the dielectric function for RuO_2_, Ta_2_O_5_, and WO_3_ oxide layers, showing an overall dielectric behavior post-high-temperature cycle. Insets in (b) and (d) show real-color photographs of the samples before and after high-temperature treatment at near-normal incidence (area 4 mm × 4 mm).

Given the change in surface composition identified by XPS and the predicted modification in optical behavior from Figure 1, we analyze the newly formed metal oxide layers by measuring their dielectric functions at room temperature before and after the heating cycle. Figure 3 presents the optical properties of the three refractory metals (Ru, Ta, and W), measured *via* spectroscopic ellipsometry. The dielectric function of all three metals is determined using the general-oscillator model (tables of model parameters for metals and oxides are available in Tables S1 and S2 in the Supporting Information, respectively). We fabricate the thin films by sputtering onto a standard Si wafer and onto a reference glass substrate. By measuring transmission data from the glass reference sample included in the thin-film deposition (Figure S2 in the Supporting Information), we verify that all three metal thin films are optically thick prior to high-temperature treatment given that the intensity of transmitted light is less than 5% in all cases. All three materials exhibit strongly metallic behavior in the visible region as evidenced by their mostly negative ε_1_, and begin silver in color, as shown in the insets of Figure 3b. We observe limited oxidation prior to high-temperature treatment as evidenced by Figure 2a–d, although effects on the sample are negligible given that their behavior is still strongly metallic and reflective. These results are comparable to previous literature examples of each metal.^35−37^

As presented in Figure 3c–d, the oxide layers display an overall dielectric optical behavior, exemplified in their transparency across a wide wavelength range and their positive ε_1_. The dielectric functions line up well with previous literature sources for these oxides.^38−40^ Our model indicates the presence of a remaining metallic layer underneath two of the MO*_x_* (Ru and W); therefore, we obtain the dielectric function of the metallic and oxide layer of these structures after high-temperature treatment. Here, we observe a three-layer structure with the newly formed metal oxide acting as a top dielectric film, a metal intermediate layer, and a bottom dielectric Si substrate. For the Ta sample, oxygen diffused throughout the entire metallic layer where a Ta_2_O_5_/Si system is formed (see Figure S3 in the Supporting Information for fits to ellipsometric data, along with the calculated thicknesses of each layer). Since Ta was fully oxidized, the dielectric function for Ta presented in Figure 3a–b is determined using the pre-high-temperature reflectivity data for the sample (see Figure S4 in the Supporting Information for fit to pristine Ta/Si ellipsometric data). The relevance in accurately determining the dielectric functions of these oxides lies in using this information to realistically design structural color pixels for printing in high-temperature settings, not possible with conventional coin-age metals. Overall, the control of the thickness of both metal and MO*_x_* films enables control over the light interference within the structure, which produces vivid coloration in all three samples, enabling vibrant reflected colors as displayed in the inset of Figure 3d.

**Figure 4:**
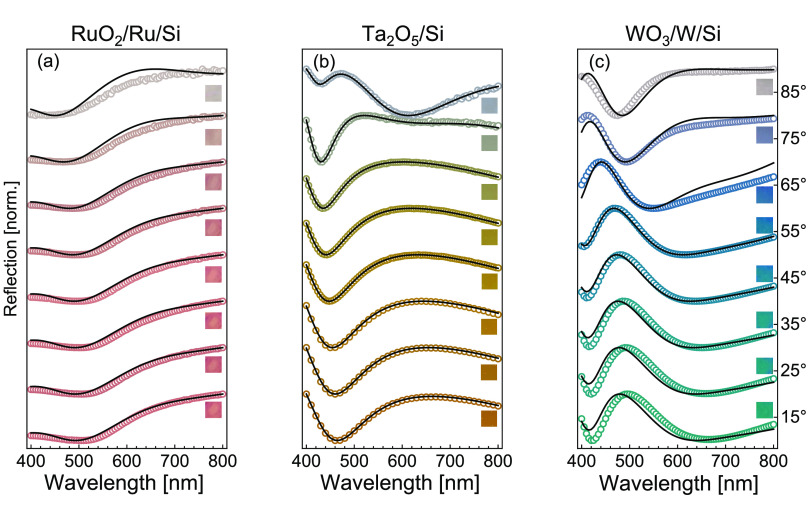
Angular dependence of chromaticity. Measured (open circles) and simulated (solid black curve) reflection spectra of structural color filters for (a) RuO_2_/Ru/Si, (b) Ta_2_O_5_/Si, and (c) WO_3_/W/Si, as the orientation of the incident light varies from 15° (nearly normal incidence) to 85°. All samples show very bright colors for a wide-angle range. The insets are real-color photographs of the samples’ surface at each angle (with area 4 mm × 4 mm).

An important feature for color pixels is chromaticity and angular insensitivity. Thus, we quantify the changes in hue as a function of light angular incidence for all pixels by measuring the reflection of each heat-treated sample every 10°. We plot the reflectivity for each system in Figure 4, from 15 to 85° from normal incidence for the visible wavelength range (see Figure S5 for full range comparison and Figure S6 for full reflection maps). The data are normalized at each angle such that each curve has a minimum at 0 and a maximum at 1. All three samples show bright coloration for a wide range of angle values. The reflectivity of all three structures is angle-insensitive up to at least 65° as has been previously demonstrated from thin-film-interference-based structural color or superabsorber systems,^2^ demonstrating the potential for these materials as wide-angle visible reflectors for structural color applications.

**Figure 5:**
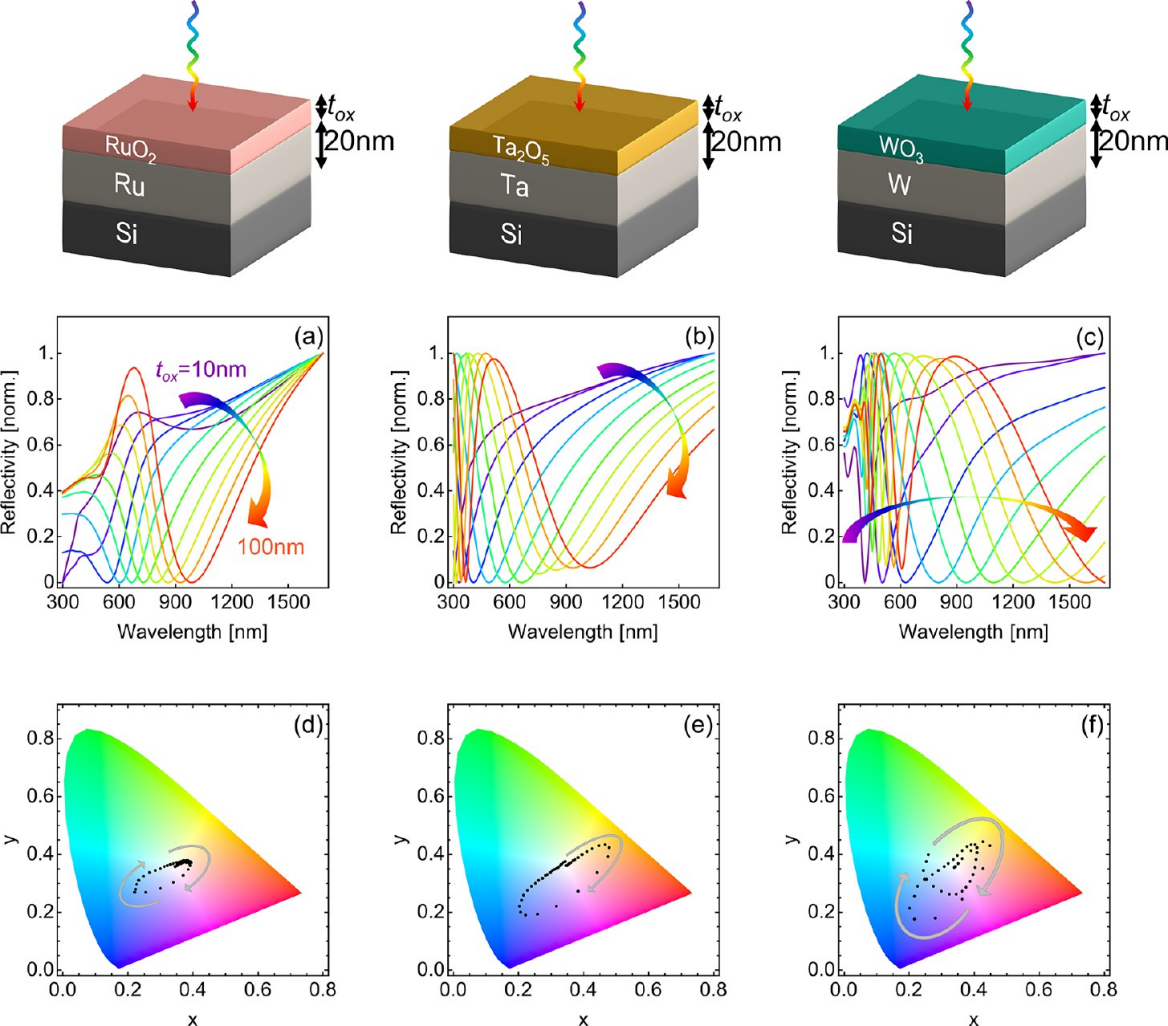
Multiwavelength reflectors for color printing at elevated temperatures. Top row: device schematics for post-high-temperature-treated structures. (a–c) Calculated reflection spectra for (a) RuO_2_/Ru/Si, (b) Ta_2_O_5_/Ta/Si, and (c) WO_3_/W/Si as the thickness of the refractory metal oxide layer varies from 10 nm to 100 nm in steps of 10 nm. (d–f) Color gamut for calculated reflection spectra for (d) RuO_2_/Ru/Si, (e) Ta_2_O_5_/Ta/Si, and (f) WO_3_/W/Si using experimental dielectric functions for all materials, as the thickness of the refractory metal oxide layer varies from 0 to 100 nm in steps of 5 nm.

With the dielectric function of each metal and its MO*_x_* counterpart, we simulate the expected reflection performance for different values of oxide layer thickness on top of a 20 nm metal layer using the transfer matrix method (TMM) (see top row of Figure 5 for schematics).^41^Figure 5a–c shows the calculated normal-incidence reflection spectra for different thicknesses of the metal oxide layer *t*_ox_, varying from 10 to 100 nm in steps of 10 nm. For all three metals, the reflection characteristics reliably shift to longer wavelengths as the oxide thickness increases, suggesting that pixels across a wide range of the color gamut should be fabricable simply by changing the oxide layer thickness, which can be controlled by varying the length of time a sample is held at 600 °C. Figure 5d–f shows the chromaticity diagrams for the simulated structures for our three samples as the thickness of the refractory metal oxide layer varies from 0 to 100 nm in steps of 5 nm. As one can observe, the color ranges across a large region of the color gamut simply by increasing the thickness of the oxide layer. The highly tailorable reflectivity and chromaticity achievable with a three-layer reflector geometry, as demonstrated in Figure 5, highlight these materials’ promise as photonic active components for high-temperature applications.

**Figure 6:**
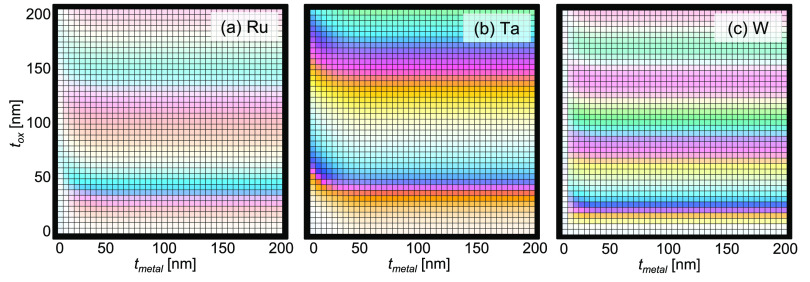
Simulated color pixels for MO*_x_*/M/Si stacks. Simulated colors of (a) Ru, (b) Ta, and (c) W varying both the metal thickness (*t*_metal_) and the oxide thickness (*t*_ox_) from 0 nm to 200 nm in steps of 5 nm.

Next, we calculate the color of different possible pixels by varying both the metal and the oxide thickness using the simulated reflectivity at normal incidence.^42^Figure 6 shows the simulated color for our three materials for film thicknesses ranging from 0 nm to 200 nm in steps of 5 nm. When varying both thicknesses, we can achieve very vivid coloration across a large portion of the color gamut. As seen in Figure 6a, Ru-based samples present overall pastel shades, as a direct consequence of their wider reflectance spectra as in Figure 4a. Conversely, Ta_2_O_5_/Ta/Si and WO_3_/W/Si both offer bright color options throughout most of the visible spectrum due to the narrower peaks in the visible region of their reflectivity spectra. These simulations show the promise of refractory metal oxides for industry-scalable structural color pixels with controllable high-temperature behavior (offering either static or reversible response, depending on material selection), with options ranging from pale to bright colors across the majority of the visible color spectrum. The sharp changes in color with very small changes in thickness also promote one possible use for this structure: in high-temperature applications requiring very low levels of oxygen, these quickly oxidizing samples can serve as highly sensitive oxygen sensors, in which a color change could quickly detect the presence of oxygen.

While all three structures are formed of materials with melting points >1100 °C, the oxides present distinct thermochemical properties. Ta_2_O_5_ has previously been demonstrated to remain stable in inert environments at temperatures beyond 1000 °C,^20^ while the other two oxides (RuO_2_ and WO_3_) have been shown to reduce to their pure constituent metals beyond 800 °C.^43−45^ Thus, the unique material chemistry of each metal oxide is a feature: RuO_2_ or WO_3_ can be implemented in situations that require color reversibility, while Ta_2_O_5_ is the best choice to attain high-temperature stability. For applications in oxygen sensing, reusability is a highly desirable trait. With the reversibility of the oxidation process for RuO_2_ and WO_3_ thin films, oxygen sensors formed using Ru and W oxide thin films would be fully reusable after reannealing the oxidized film in an inert environment (Ar or N_2_). In contrast, the oxidation of Ta_2_O_5_ being irreversible presents benefits for applications requiring stable coloration, for example as conformal coatings for space applications.

## Conclusions

In summary, we realized a platform for structural color filters that can operate at temperatures beyond 1100 °C, based on refractory metals and their oxides. We validated the suitability of these materials by determining the changes in their optical properties upon heating treatments in an oxidizing environment. As an example, we demonstrated vibrant hues across a wide portion of the color gamut by submitting Ru, Ta, and W to identical thermal treatments at 600 °C. The development of a metal oxide dielectric layer produced interference that led to vivid colors. The refractory, dielectric layers required for interference are achieved using *in situ* oxidation, a reaction that can be reversible or not, depending on the metal and medium. A distinctive aspect of our approach is the promise of these structures at high temperatures: given their high melting points and differing thermochemical behavior, these structures offer tailorable chromaticity and material-dependent reversibility (RuO_2_, WO_3_) or static optical behavior (Ta_2_O_5_) upon high-temperature treatment in inert environments. Furthermore, our oxide growth method allows for very precise control of dielectric layer thickness *via in situ* optical measurements, which can determine the thickness in real time as the oxide layer grows. Overall, these results show the potential of refractory metals for photonics under extreme conditions and how oxidation can be implemented as a powerful route to attain dielectric layers *in situ*, which can work as optical markers at elevated temperatures.

## Experimental Methods

### Sample Fabrication

Samples were fabricated *via* DC magnetron sputtering on a Kurt J. Lesker PVD 200 sputterer. All depositions were in an inert environment (Ar). Deposition parameters for each material are shown in Table S3. The metals were deposited onto a standard Si wafer and onto glass, as a reference.

### In Situ Ellipsometry

*In situ* ellipsometry results were measured on a J. A. Woollam VASE ellipsometer, with a Linkam RC-2 heating stage providing high-temperature control up to 600 °C. Samples were heated from room temperature (25 °C) to 600 °C with a ramping rate of 3 °C min^–1^, with holds at every 100 °C to allow the sample to thermalize and to allow for detailed ellipsometric measurements. Above 400 °C, we also stop every 50 °C to allow for finer visualization of the high-temperature behavior of the samples. The full temperature profile is shown in Figure S1 in the Supporting Information, along with real-color photographs of each sample before and after high-temperature treatment.

### Ex Situ Optical Measurements and Simulations

The *ex situ* ellipsometry measurements were taken on a J. A. Woollam M-2000 ellipsometer (193–1688 nm). Dielectric functions are determined by fitting the ellipsometric parameters Ψ and Δ, fitting with general-oscillator models for both the pure metals and the oxides after high-temperature treatment using the CompleteEASE software. The individual oscillators used for each model are shown in Table S1 in the Supporting Information, using the standard equations for each given in CompleteEASE.^28^ To confirm that the samples were optically thick prior to high-temperature treatment, transmission and reflection data were measured from samples deposited on glass in the same deposition run; transmission measurements on each sample were compared to a straight-through baseline in air. Reflectivity measurements were taken on a J. A. Woollam W-VASE ellipsometer (290–2440 nm). The optical simulations showing the reflection as a function of changing oxide thickness, and the simulated color as a function of changing metal and oxide thicknesses, were simulated in CompleteEASE using the thicknesses and dielectric functions that were determined using *ex situ* ellipsometry.

### X-ray Photoelectron Spectroscopy (XPS)

XPS measurements were taken on a Kratos SUPRA Axis XPS with a monochromated Al Kα source (1486.6 eV). The chamber’s base pressure was 2 × 10^–8^ Torr, with a 7 mA emission current and a scan size of 450 × 900 μm. Peaks were fitted using Kratos ESCApe; normalization and Shirley background subtraction were performed after fitting.

## Supporting Information Available

Tabulated dielectric functions for metals and oxides from paper, as well as Mo and MoO_3_ (TXT)
Real-color photographs of samples, temperature profile of high-temperature treatment, parameters for dielectric functions of metals and oxides, transmission measurements for thin films deposited on glass, experimental Ψ and Δ before and after high-temperature treatment compared to model fit, extended reflectivity plots and reflection maps, extended *in situ* ellipsometry measurements, sputter deposition parameters, *in situ* ellipsometry plots for Mo presented as the fourth sample of study (not included in the main text due to lower melting point of MoO_3_), XPS narrow scan for Mo and MoO_3_, reflection spectra for Mo, reflection and chromaticity simulations for Mo, and extended plots for Mo (PDF)
