# Supplementary material for: Refractory Metals and Oxides for High-Temperature Structural Color Filters
Source: ACS Appl Mater Interfaces. 2022 Dec 6;14(50):55745–52. doi: 10.1021/acsami.2c14613 (PMC9782350; doi:10.1021/acsami.2c14613)
Supplement: Supplementary file 2 — am2c14613_si_002.pdf [file am2c14613_si_002.pdf]

**Supporting Information**

# Refractory metals and oxides for high-temperature structural color filters

*Margaret A. Duncan*[1], *Landin Barney*[2], *Mariama Rebello Sousa Dias*[2], *and Marina S. Leite*[1]*

[1]Department of Materials Science and Engineering, UC Davis, 1 Shields Ave, 95616, USA

[2] Department of Physics, University of Richmond, 138 UR Drive, 23173, USA

*E-mail: mleite@ucdavis.edu

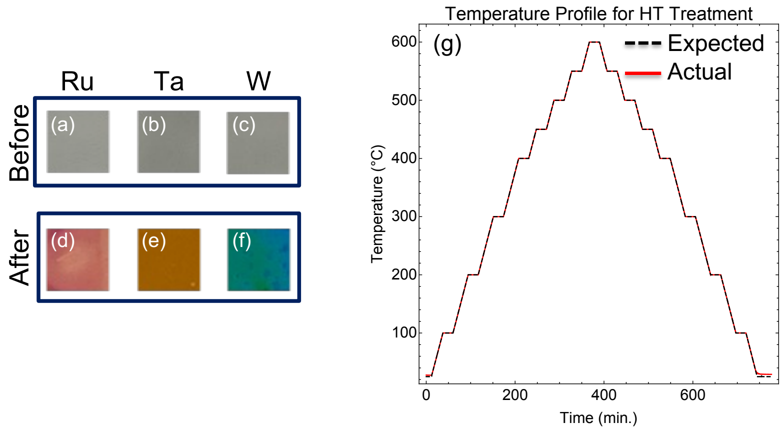


**Figure S1:** Real-color photographs of (a) Ru, (b) Ta, and (c) W samples before high temperature treatment, and (d) $RuO_2$, (e) $Ta_2O_5$, and (f) $WO_3$ after heating treatment, under similar illumination conditions and camera angle. (g) Temperature profile for all annealing treatments. In all cases, the chamber environment was a mixture of Ar and $O_2$. We use a ramping rate of 3°C/min, and stop at each 100°C for a total of 22 min to allow the samples to thermalize. At higher temperatures (>400°C), we stop for 22 min every 50°C to allow the samples to thermalize.

**Table S1:** Dielectric functions for (a) Ru, (b) Ta, and (c) W.

**(a) Ru Parameters ($E_\infty = 0$)**

| Oscillator Type | ρ (Ω·cm) | τ (fs) | |
|---|---|---|---|
| **Drude** | $6.651\times10^{-5}$ | 1.805 | - |
| **Oscillator Type** | **Amplitude** | **Broadness** | **Energy** |
| **Sellmeier** | -564.1585 | - | 8.690 |
| **Lorentz** | 321.42692 | 0.2736 | 9.168 |
| **Lorentz** | 6.667426 | 0.7216 | 1.818 |
| **Lorentz** | 18.935020 | 4.1704 | 2.480 |

**(b) Ta Parameters ($E_\infty = 2.5362$)**

| Oscillator Type | Amplitude | Broadness | Energy |
|---|---|---|---|
| **Lorentz** | 419.95 | 0.5579 | 0.1869 |
| **Gaussian** | 7.8647 | 1.072 | 0.2286 |
| **Gaussian** | 9.913 | 2.4654 | 1.0221 |
| **Gaussian** | 3.17 | 3.4942 | 3.6899 |
| **Gaussian** | 8.6764 | 8.6842 | 2.0749 |
| **Gaussian** | 1.5144 | 8.6842 | 2.0749 |
| **Gaussian** | 0.89004 | 0.46966 | 3.417 |

**(c) W Parameters ($E_\infty = 7.0951$)**

| Oscillator Type | ρ (Ω·cm) | τ (fs) | |
|---|---|---|---|
| **Drude** | $2.3084\times10^{-5}$ | 3.5096 | - |
| **Oscillator Type** | **Amplitude** | **Broadness** | **Energy** |
| **Gaussian** | 22.42 | 0.26419 | 0.63752 |
| **Gaussian** | 58.427 | 1.031 | 2.4064 |
| **Gaussian** | 75.198 | 1.4946 | 0.83888 |
| **Gaussian** | 53373 | 0.00754 | 18.871 |

**Table S2:** Fit parameters for dielectric functions of (a) $RuO_2$, (b) $Ta_2O_5$, and (c) $WO_3$.

### (a) $RuO_2$ Parameters ($E_\infty = 1$)

| Oscillator Type | ρ (Ω·cm) | τ (fs) | | |
|---|---|---|---|---|
| **Drude** | 0.001 | 5 | - | - |
| **Oscillator Type** | **Amplitude** | **Broadness (eV)** | **Energy (eV)** | **BG Energy (eV)** |
| **Tauc-Lorentz** | 11.4039 | 1.096 | 2.807 | 1.032 |
| **Tauc-Lorentz** | 22.3271 | 3.926 | 5.023 | 1.092 |
| **Tauc-Lorentz** | 2.4012 | 1.043 | 3.620 | 1.061 |
| **Tauc-Lorentz** | 3.5733 | 1.072 | 6.583 | 0.976 |

### (b) $Ta_2O_5$ Parameters ($E_\infty = 2.5362$)

| Oscillator Type | Amplitude | Broadness (eV) | Energy (eV) | BG Energy (eV) |
|---|---|---|---|---|
| **Sellmeier** | 158.05 | - | 10.525 | - |
| **Tauc-Lorentz** | 211.75 | 1.8544 | 5.2103 | 4.0505 |
| **Gaussian** | 0.11219 | 10 | 4.185 | - |

### (c) $WO_3$ Parameters ($E_\infty = 1$)

| Oscillator Type | Amplitude | Broadness (eV) | Energy (eV) | BG Energy (eV) |
|---|---|---|---|---|
| **Tauc-Lorentz** | 149.84 | 0.49105 | 3.468 | 2.5074 |
| **Gaussian** | 28.396 | 2.8234 | 5.6561 | - |

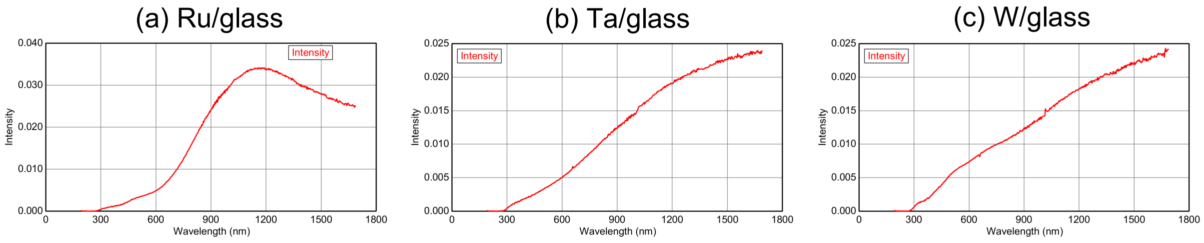


**Figure S2:** Transmission data for (a) Ru, (b) Ta, and (c) W deposited on glass, during same deposition run as thin films on silicon. Given the low magnitude of transmitted intensity (<4% for all samples), we can confirm that the thin films are optically thick for all three materials.

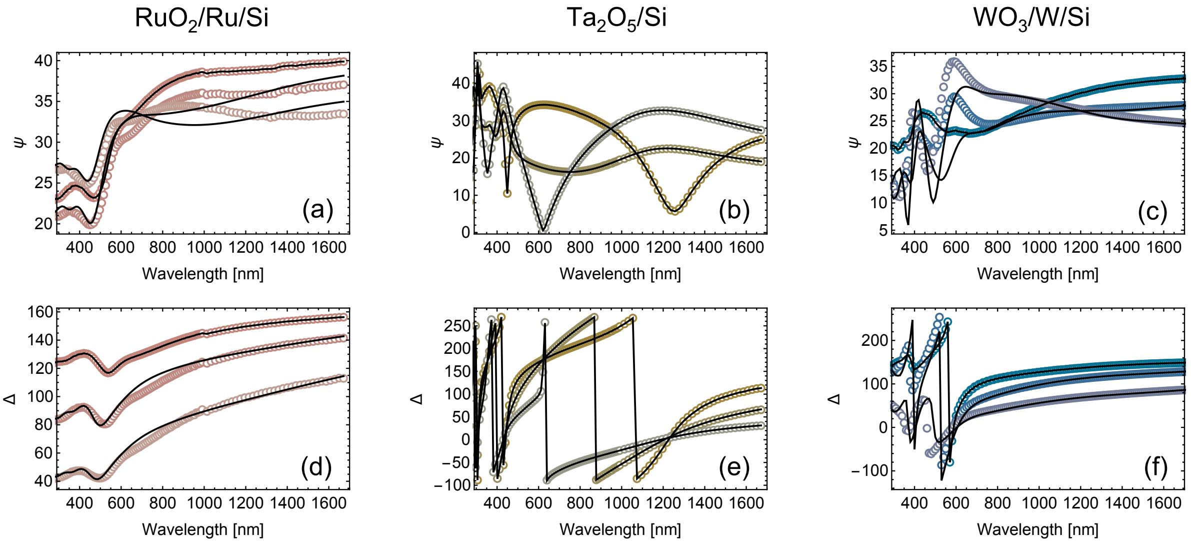


**Figure S3:** Measured (open circles) and fitted (black lines) (a-c) Ψ and (d-f) Δ for (a,d) $RuO_2$/Ru/Si, (b,e) $Ta_2O_5$/Ta/Si, and (c,f) $WO_3$/W/Si, obtained at three different angles (60°, 65°, and 70°) and fit using General Oscillator models. The thicknesses for the metal oxide and metal layers were determined to be 32.40 nm and 20.01 nm for $RuO_2$ and Ru, 151.44 nm for Ta, and 7.356 nm and 21.955 nm for $WO_3$ and W. The models for the Ta and W systems also include an EMA roughness layer made up of 50% void and 50% oxide, with thicknesses of 2.240 nm and 41.418 nm for Ta and W, respectively.

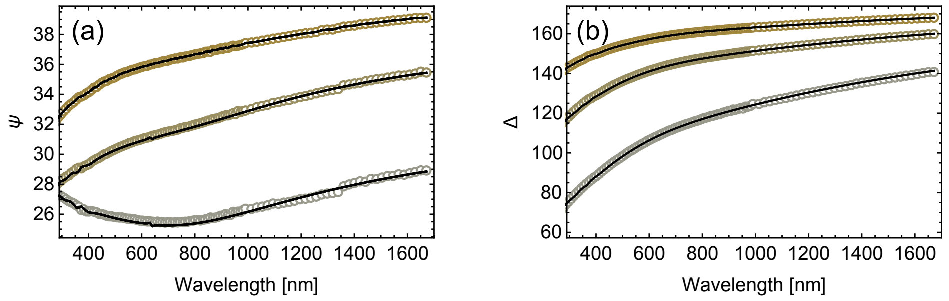


**Figure S4:** Ellipsometric parameters (a) Ψ and (b) Δ for Ta for three different angles of incidence (55°-75°), for use in simulations. Model is fit using data for each material's determined dielectric functions, and is shown with the solid black lines. Experimental data is shown in colored circles, corresponding to the colors of the samples at each respective angle.

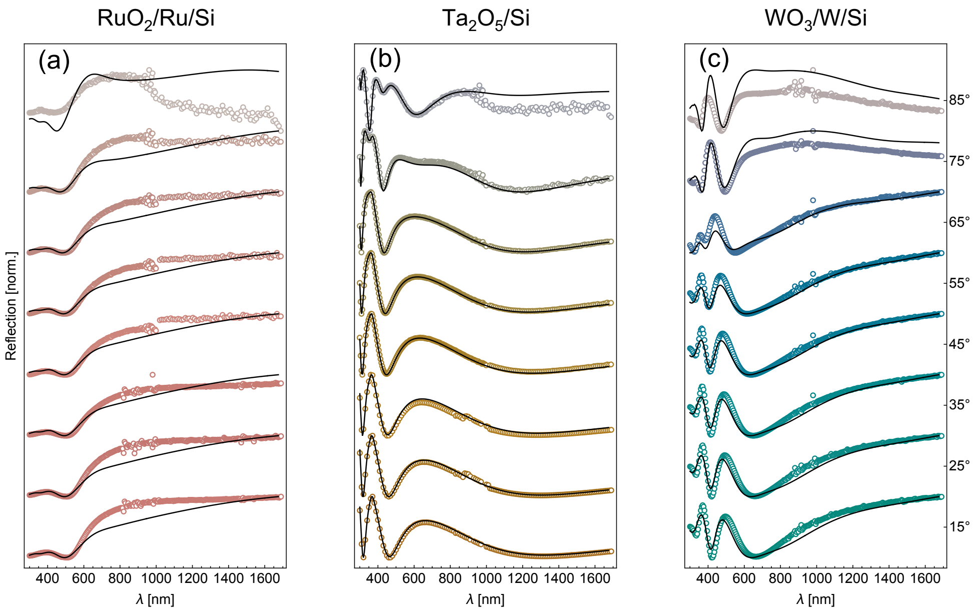


**Figure S5:** Angular dependence of chromaticity, extended to full wavelength range for reference. Measured (open circles) and simulated (solid black curve) reflection spectra of structural color filters for (a) $RuO_2$/Ru/Si, (b) $Ta_2O_5$/Ta/Si, and (c) $WO_3$/W/Si, as the orientation of the incident light varies from 15° (nearly normal incidence) to 85°. Fits demonstrate high accuracy from 15°-75°; inaccuracies in 85° can be attributed to noise in reflectivity measurements past 1000 nm (due to switch in detectors around 1000 nm). Note: noise is corrected in ellipsometry data (Figs S3 and S4) since Ψ measures the ratio of polarized light intensity and Δ measures phase difference. Thus, ellipsometry data is more trustworthy than reflection data past 1000 nm.

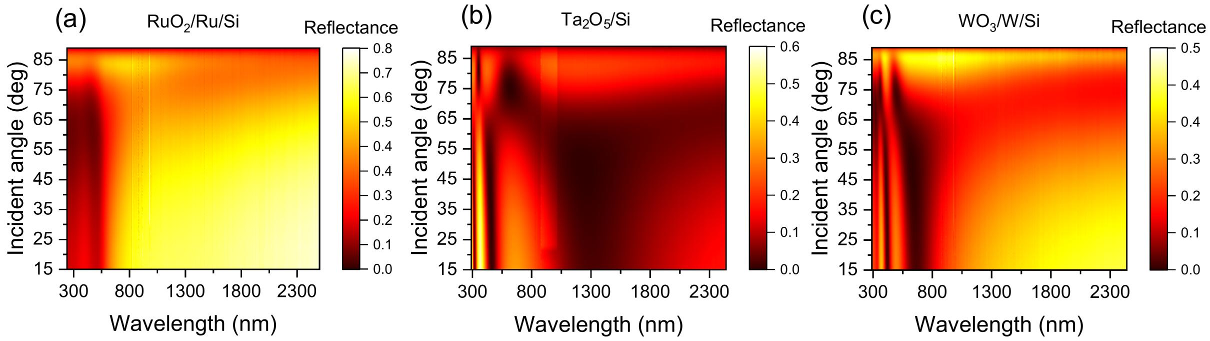


**Figure S6:** Reflection maps of (a) $RuO_2$/Ru/Si, (b) $Ta_2O_5$/Si, and (c) $WO_3$/W/Si after high-temperature treatment, from 15° to 89°, acquired every 0.2°.

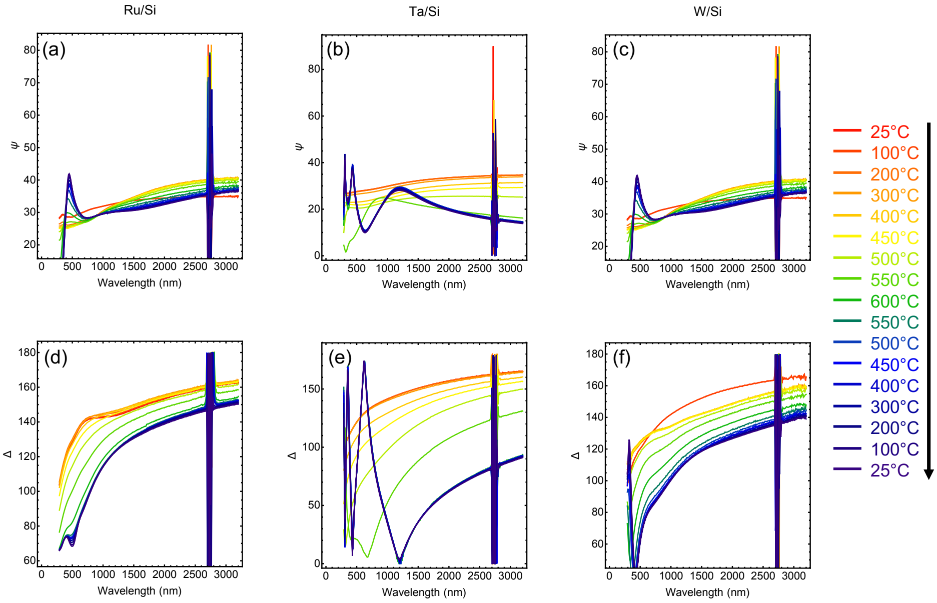


**Figure S7:** (a-c) Ψ and (d-f) Δ for (a, d) Ru/Si, (b, e) Ta/Si, and (c, f) W/Si as temperature changes from room temperature to 600°C. Discontinuity around 2750 nm is due to window glass on environmental chamber of the ellipsometer.

**Table S3:** Deposition parameters for DC magnetron sputtering in Ar environment of Ru, Ta, and W utilizing a Lesker PVD200 sputtering system.

| Material | Power (W) | Pressure (mTorr) | Time (sec) |
|---|---|---|---|
| **Ru** | 200 | 3 | 1800 |
| **Ta** | 200 | 3 | 500 |
| **W** | 200 | 3 | 500 |

A fourth sample based on a Mo thin film was fabricated and studied in the same detail as the ones presented in the main text, though it was not included in the main text due to its lower oxide melting point; the figures for this extra sample are available in Figures S8 – S14, with deposition parameters and optical fit parameters for the structure detailed in Table S4.

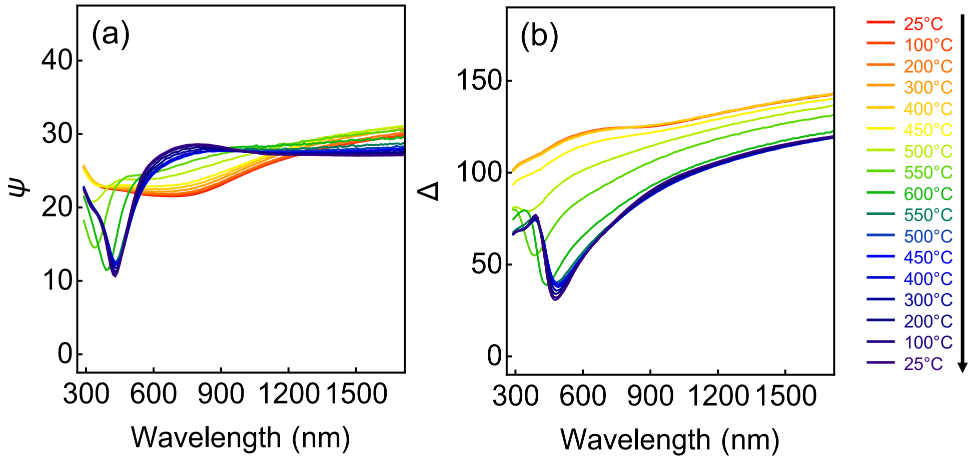


**Figure S8:** *In situ* measurements of (a) Ψ and (b) Δ at high temperature for Mo/Si. With these two parameters, we can characterize the optical properties of these materials as they change with increasing temperature. All curves shown are at an angle of 70° from normal incidence. Black arrow represents order of measurements, from initial to final.

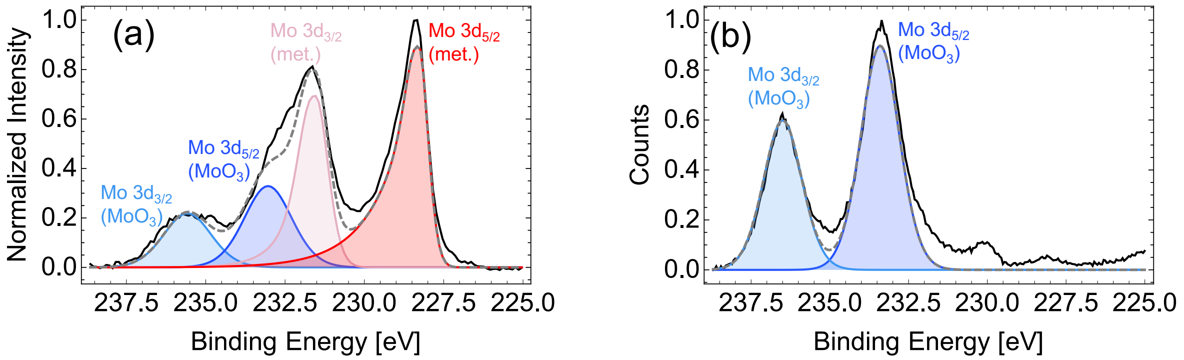


**Figure S9:** XPS narrow-scan measurement of Mo 3d peak (a) before and (b) after high-temperature treatment. In each plot, the black-solid and grey-dashed lines refer to raw data and their respective fits using the contributions of all peaks in blue and red. The chemical compositions of each constituent peak are shown in the plots for reference. These peaks are consistent with prior literature results for Mo thin films with a thin native oxide layer[1] and a $MoO_3$ thin film[2] before and after high-temperature treatment, respectively.

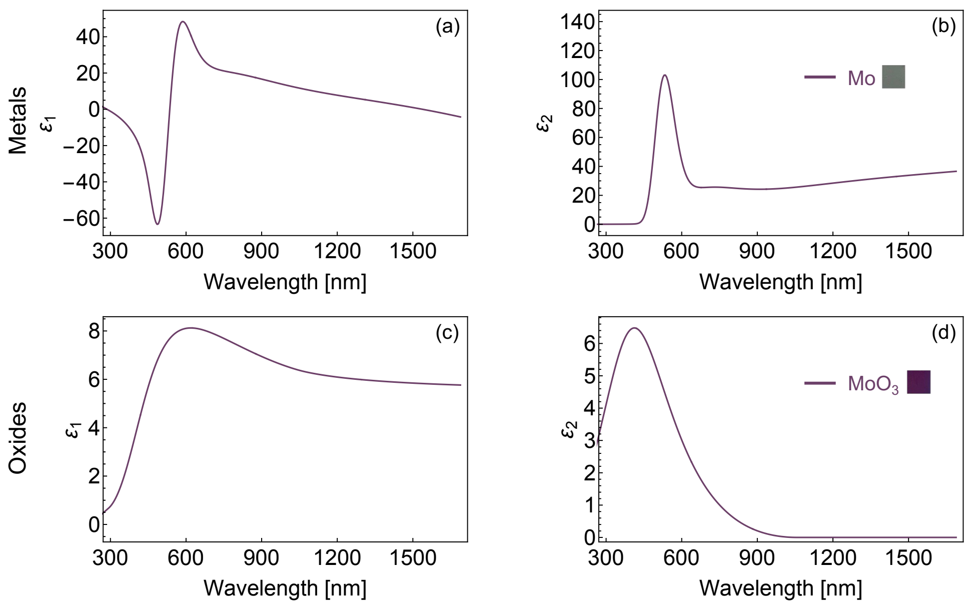


**Fig. S10:** Optical behavior of refractory metals and their oxides. (a) Real ($\varepsilon_1$) and (b) imaginary ($\varepsilon_2$) components of the dielectric function of Mo thin film, showing metallic behavior. (c) Real and (d) imaginary components of the dielectric function for $MoO_3$ oxide layer, showing overall dielectric behavior. Insets of (b) and (d) show real-color photographs of the samples before and after high-temperature treatment at near-normal incidence (area 4 mm x 4 mm). The dielectric functions of Mo and $MoO_3$ are comparable with previous literature results for both materials[3].

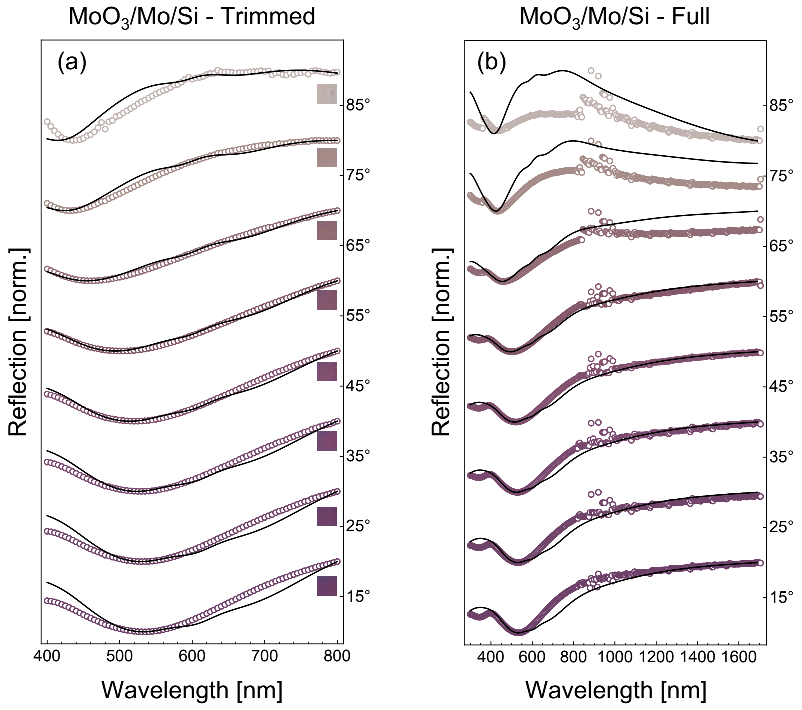


**Figure S11:** (a) Trimmed and (b) full range reflection spectra for $MoO_3$/Mo/Si sample after high-temperature treatment, from 15° to 85°. Insets of (a) are real-color photographs of the samples' surface at each angle (with area 4 mm x 4 mm).

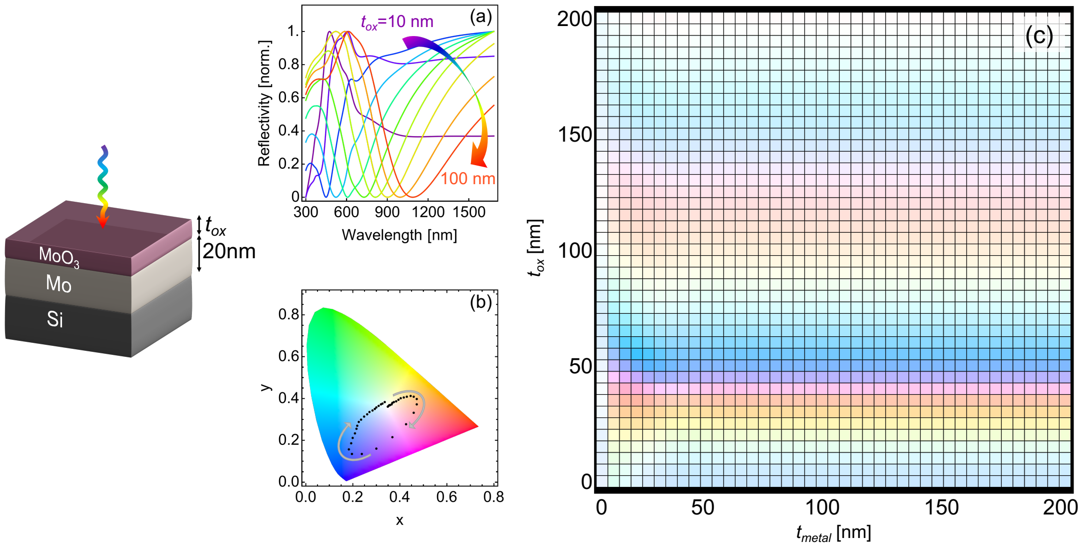


**Figure S12:** Photographs of Mo-based sample (a) before and (b) after high-temperature treatment, with area 4 mm x 4 mm. (c) Transmission data for Mo deposited on glass, confirming low enough transmission to consider films optically thick. Measured (open circles) and fitted (black lines) (d) ) Ψ and (e) Δ for $MoO_3$/Mo/Si, obtained at three different angles (60°, 65°, and 70°) and fit using General Oscillator models. The thicknesses for the metal oxide and metal layers were determined to be 41.92 nm and 26.54 nm for $MoO_3$ and Mo, respectively.

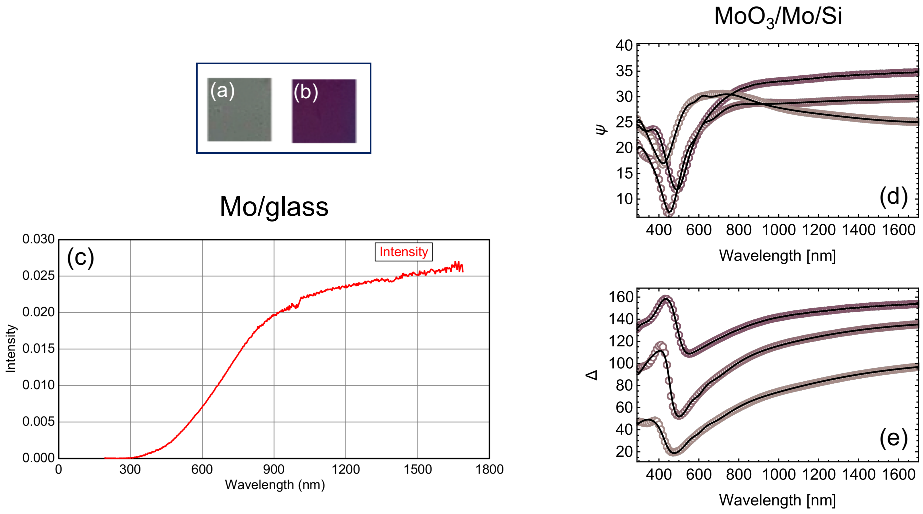


**Figure S13:** Multi-wavelength superabsorbers for color printing at elevated temperatures. Left: device schematic for post-high-temperature treated structure. (a) Calculated reflection spectra for $MoO_3$/Mo/Si as the thickness of the refractory metal oxide layer varies from 10 nm to 100 nm in steps of 10 nm. (b) Color gamut for calculated reflection spectra for $MoO_3$/Mo/Si using experimental dielectric functions, as the thickness of the refractory metal oxide layer varies from 0 nm to 100 nm in steps of 5 nm. (c) Simulated colors of Mo varying both the metal thickness ($t_{metal}$) and the oxide thickness ($t_{ox}$) from 0 nm to 200 nm in steps of 5 nm.

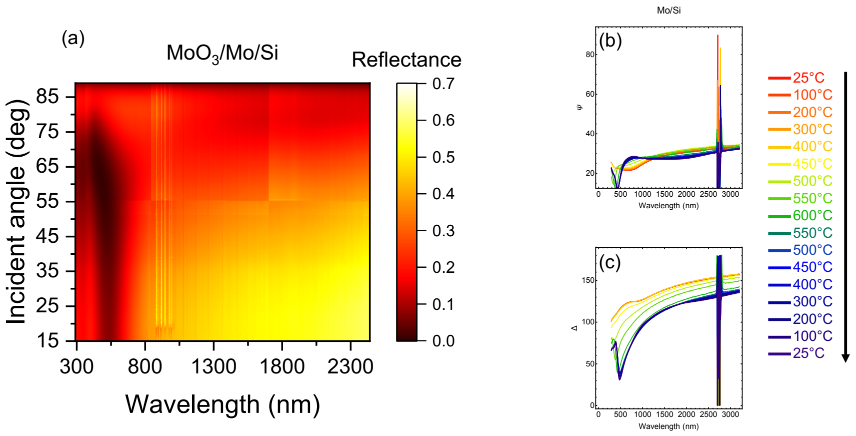


**Figure S14:** (a) Reflection map $MoO_3$/Mo/Si after high-temperature treatment, from 15° to 89° degrees. We take measurements every 0.2°. Measurements taken using a J. A. Woollam W-VASE Ellipsometer. Ellipsometric parameters (b) Ψ and (c) Δ for Mo/Si as temperature changes from room temperature to 600°C. Discontinuity around 2750 nm is due to window glass on measurement chamber.

**Table S4:** Molybdenum sample (a) metal dielectric function parameters, (b) oxide dielectric function parameters, and (c) sputter deposition parameters for DC magnetron sputtering in an Ar environment. Sputtering performed on a Lesker PVD200 sputtering system.

**(a) Mo Parameters ($E_\infty$ = 8.2397)**

| Oscillator Type | ρ (Ω·cm) | τ (fs) | |
|---|---|---|---|
| **Drude** | $5.832\times10^{-5}$ | 3.4916 | - |
| **Oscillator Type** | **Amplitude** | **Broadness** | **Energy** |
| **Gaussian** | 37.549 | 0.48387 | 1.5669 |
| **Gaussian** | 96.042 | 2.3362 | 0.39715 |
| **Gaussian** | 19.045 | 1.8158 | 0.80696 |

**(b) $MoO_3$ Parameters ($E_\infty$ = 1)**

| Oscillator Type | Amplitude | Broadness (eV) | Energy (eV) | BG Energy (eV) |
|---|---|---|---|---|
| **Sellmeier** | 26.388 | - | 5.7121 | - |
| **Tauc-Lorentz** | 39.587 | 2.419 | 3.0281 | 1.1386 |

**(c) Mo Sputter Deposition Parameters**

| Quantity | Value |
|---|---|
| **Power (W)** | 200 |
| **Pressure (mTorr)** | 3 |
| **Time (sec)** | 500 |

**References:**

(1) Vos, M. F. J.; Macco, B.; Thissen, N. F. W.; Bol, A. A.; Kessels, W. M. M. (Erwin). Atomic Layer Deposition of Molybdenum Oxide from (NtBu)2(NMe2)2Mo and O2 Plasma. J. Vac. Sci Technol., A 2016, 34 (1), 01A103. https://doi.org/10.1116/1.4930161.

(2) Široký, P.; Sobota, J.; Seidl, J.; Jastrabík, L. Ellipsometry of Ultrathin Mo and Si Films. Thin Solid Films 1993, 234 (1), 500–502. https://doi.org/10.1016/0040-6090(93)90317-I.

(3) Baltrusaitis, J.; Mendoza-Sanchez, B.; Fernandez, V.; Veenstra, R.; Dukstiene, N.; Roberts, A.; Fairley, N. Generalized Molybdenum Oxide Surface Chemical State XPS Determination via Informed Amorphous Sample Model. Appl. Surf. Sci. 2015, 326, 151–161. https://doi.org/10.1016/j.apsusc.2014.11.077.
